# Results of the SEL-I-METRY Phase II Trial on Resensitization of Advanced Iodine Refractory Differentiated Thyroid Cancer to Radioiodine Therapy

**DOI:** 10.1089/thy.2022.0707

**Published:** 2023-09-13

**Authors:** Jon Wadsley, Gemma Ainsworth, Amy Beth Coulson, Kate Garcez, Laura Moss, Kate Newbold, Kate Farnell, Jayne Swain, Helen Howard, Matthew Beasley, Andrew Weaver, Katie Wood, Jennifer Marshall, Matthew Griffin, Abigail Pascoe, Yong Du, Jan Taprogge, Glenn Flux, Sarah Brown

**Affiliations:** ^1^ Weston Park Hospital, Sheffield, United Kingdom.; ^2^ Leeds Cancer Research UK Clinical Trials Unit, Leeds Institute of Clinical Trials Research, University of Leeds, Leeds, United Kingdom.; ^3^ Christie NHS Foundation Trust, Manchester, United Kingdom.; ^4^ Velindre Cancer Centre, Cardiff, United Kingdom.; ^5^ The Royal Marsden NHS Foundation Trust, London, United Kingdom.; ^6^ Butterfly Thyroid Cancer Trust, NCCC Freeman Hospital, Newcastle, United Kingdom.; ^7^ Bristol Haematology and Oncology Centre, Bristol, United Kingdom.; ^8^ Oxford Churchill Hospital, Oxford, United Kingdom.; ^9^ Royal Surrey County Hospital, Guildford, United Kingdom.; ^10^ University Hospital Southampton, Southampton, United Kingdom.; ^11^ Nottingham University Hospitals NHS Trust, Nottingham, United Kingdom.; ^12^ Joint Department of Physics, Royal Marsden NHS Foundation Trust, Sutton, United Kingdom.; ^13^ The Institute of Cancer Research, London, United Kingdom.

**Dear Editor:**

Radioactive iodine (RAI) refractory differentiated thyroid cancer (DTC) carries a poor prognosis with a 10-year survival rate of only 10%.^1^ Recently, multitargeted tyrosine kinase inhibitors have been tested in patients with RAI refractory DTC. Two phase III trials demonstrated improvements in progression-free survival (PFS): DECISION^2^ showed improvement from 5.8 to 10.8 months with sorafenib, SELECT^3^ demonstrated improvement from 3.6 to 18.3 months with lenvatinib. However, both agents cause significant toxicities. Treatment is continuous until disease progression, which can significantly affect quality of life.

Activation of the MAP kinase signaling pathway can lead to reduced expression of the sodium iodide symporter. Selumetinib (ARRY-1428860) is an oral, potent, and highly selective allosteric MEK1/2 inhibitor that can block inappropriate signaling through this pathway and potentially restore iodine sensitivity.^4^

A previously reported pilot study^5^ tested selumetinib 75 mg twice daily for 4 weeks in patients with RAI refractory DTC. Of 20 patients evaluated, selumetinib increased iodine uptake (IU) in 12 patients. Of these, eight achieved sufficient uptake for further RAI, according to predefined criteria. Five of these patients had partial response and three stable disease. All had decreases in serum thyroglobulin and no grade ≥3 adverse events (AEs) were noted.

SEL-I-METRY was a single-arm multicenter phase II trial, assessing whether selumetinib 75 mg twice daily for 28 days followed by ^131^I therapy leads to increased PFS in patients demonstrating increased ^123^I IU after selumetinib.

Full trial details are reported previously.^6,7^ Key eligibility criteria included measurable RAI refractory DTC that had progressed within 12 months by RECIST v1 · 1.^8^ Disease was considered RAI refractory if ≥1 measurable lesion did not demonstrate IU on a previous RAI scan, or if ≥1 measurable lesion had progressed within 12 months of RAI, despite demonstrable radioiodine avidity at the time of treatment.

Patients with prior exposure to tyrosine kinase, MEK, RAS, or RAF inhibitors, those who required high-iodine content medication, or those with an iodine contrast-enhanced CT scan within two months before trial enrolment were excluded.

The study was approved by East Midlands, Leicester South Research Ethics Committee (15/EM/0455) and the Medicines and Healthcare Products Regulatory Agency, and is registered on ISRCTN (ISRCTN17468602). All patients provided written informed consent.

All participants were planned to receive 28 days oral selumetinib (75 mg twice daily). RAI uptake at baseline and 28 days was assessed through ^123^I SPECT/CT scan after 2 administrations of recombinant human thyrotropin (rhTSH) (0.9 mg intramuscular administration daily for 2 days before imaging). Imaging was assessed centrally to determine suitability for further RAI therapy. Participants had sufficient RAI uptake if there was any clinically relevant uptake in lesions that had shown no uptake at baseline, or at least 30% increase in RAI uptake in a lesion with evidence of some uptake at baseline. During central review, participants continued 75 mg selumetinib twice daily (maximum 18 days).

Those with sufficient uptake continued selumetinib until two days after administration of RAI. ^131^I was delivered at an activity of 5.5 GBq (±10%), after 2 administrations of rhTSH. If there was insufficient uptake, selumetinib was discontinued, and no further trial treatment was given.

The primary endpoint was PFS at 12 months in patients with sufficient IU who received ^131^I treatment, assessed from date of registration to first documented evidence of disease progression by RECIST 1.1 or death. Secondary endpoints included safety and toxicity (Common Terminology Criteria for Adverse Events [CTCAE] v4 · 0) and proportion of patients with sufficient IU to receive RAI therapy.

Thirty-eight iodine-treated patients were required to demonstrate an improvement in 12-month PFS from 25%^2^ to 44% (corresponding to clinically relevant hazard ratio 0.6), using the Case and Morgan^9^ design with 10% one-sided alpha, 80% power. Assuming 60% iodine-uptake rate, demonstrated by Ho et al.^5^ for *any* uptake, required 60 patients, allowing 10% drop out.

The full analysis set included all registered participants receiving at least one dose of selumetinib. Participants who demonstrated uptake and received further RAI formed the iodine-treated (IT) cohort; remaining participants formed the non-IT cohort.

SEL-I-METRY opened to recruitment on March 28, 2017. The independent Data Monitoring and Ethics Committee (DMEC) reviewed the IU rate after 26 patients, finding a lower-than-anticipated rate of ∼40%. Due to slow recruitment, it was not feasible to expand the sample size further. In agreement with the Trial Steering Committee and DMEC, the trial closed to recruitment early on August 15, 2019, registering 30 patients. One patient was found ineligible postregistration, and one withdrew before receiving treatment. These patients were not included in analysis.

Table 1 displays baseline demographics for 28 patients in the analysis set.

**Table 1.** Baseline Demographics

	Nonradioactive iodine-treated cohort (*N* = 19)	Radioactive iodine-treated cohort (*N* = 9)	Total (*N* = 28)
Age (years)			
Median age, (range)	68.0 (48.0, 83.0)	58.0 (45.0, 78.0)	66.0 (45.0, 83.0)
Participant sex			
Male	12 (63.2%)	6 (66.7%)	18 (64.3%)
Female	7 (36.8%)	3 (33.3%)	10 (35.7%)
ECOG status			
ECOG status 0	11 (57.9%)	7 (77.8%)	18 (64.3%)
ECOG status 1	8 (42.1%)	2 (22.2%)	10 (35.7%)
Thyroid cancer subtype			
Papillary thyroid cancer	9 (47.4%)	2 (22.2%)	11 (39.3%)
Follicular thyroid cancer	10 (52.6%)	7 (77.8%)	17 (60.7%)
Total	19 (100%)	9 (100%)	28 (100%)
Stage group diagnosis			
I	1 (5.3%)	1 (11.1%)	2 (7.1%)
II	0 (0.0%)	2 (22.2%)	2 (7.1%)
III	5 (26.3%)	2 (22.2%)	7 (25.0%)
Iva	4 (21.1%)	1 (11.1%)	5 (17.9%)
IVb	1 (5.3%)	1 (11.1%)	2 (7.1%)
IVc	4 (21.1%)	1 (11.1%)	5 (17.9%)
Unknown	4 (21.1%)	1 (11.1%)	5 (17.9%)
Total	19 (100%)	9 (100%)	28 (100%)
Time from original diagnosis to registration (years)			
Mean (s.d.)	7.3 (4.82)	6.8 (7.47)	7.2 (5.66)
Median (range)	6.4 (1.9, 21.4)	4.6 (1.0, 25.9)	5.5 (1.0, 25.9)
IQR	4.2, 9.4	3.4, 6.9	3.8, 7.8
Missing	0	0	0
N	19	9	28
Thyroglobulin (μg/L): baseline			
Mean (s.d.)	1124.8 (2705.4)	1762.0 (2442.0)	1329.6 (2595.8)
Median (range)	161 (2, 11535)	742 (36, 7530)	240 (2, 11535)
IQR	58, 393	131, 2439	67, 1070
Missing	0	0	0
N	19	9	28
External radiotherapy received			
Yes	10 (52.6%)	2 (22.2%)	12 (42.9%)
No	9 (47.4%)	7 (77.8%)	16 (57.1%)
Total	19 (100%)	9 (100%)	28 (100%)
External radiotherapy site	(*N* = 10)	(*N* = 2)	(*N* = 12)
Neck	4 (40%)	0 (0%)	4 (25%)
Other sites	6 (60%)	2 (100%)	8 (75%)
Other treatment received^a^			
Yes	9 (47.4%)	6 (66.7%)	15 (53.6%)
No	10 (52.6%)	3 (33.3%)	13 (46.4%)
Total	19 (100%)	9 (100%)	28 (100%)
Median follow-up time (months)			
Median (range)	12.42 (2.5, 31.0)	21.06 (7.8, 27.5)	13.98 (2.5, 31.0)
*N*	19	9	28
Disease sites of RECIST v1.1 target lesions at baseline			
Lung	12 (50.0%)	28 (71.8%)	40 (63.5%)
Lymph node	6 (25.0%)	6 (15.4%)	12 (19.0%)
Bone	3 (12.5%)	0 (0.0%)	3 (4.8%)
Liver	1 (4.2%)	2 (5.1%)	3 (4.8%)
Thyroid bed	2 (8.3%)	1 (2.6%)	3 (4.8%)
Trachea	0 (0.0%)	1 (2.6%)	1 (1.6%)
Other^b^	0 (0.0%)	1 (2.6%)	1 (1.6%)
Total	24 (100%)	39 (100%)	63 (100%)

^a^ Other treatment received included right-sided modified neck dissection, surgery for liver metastases, total thyroidectomy, RFA ablation, thermal ablation lung metastases, completion thyroidectomy, cyroextraction and endobronchial, cyroextraction, and endobronical brachytherapy, additional radioiodine, laryngectomy, gamma knife, thyroid lobectomy, completion thyroidectomy, left thyroidectomy, and photodynamic therapy. Note some patients received more than one other therapy.

^b^ Other description of disease site: left paravertebral nodule.

ECOG, Eastern Cooperative Oncology Group.

Eleven of 28 (39.3% [CI: 21.5–59.4]) participants had evidence of sufficiently increased ^123^I uptake in ≥1 lesion. Ten patients were recommended further RAI based on increased uptake, however, one did not receive therapy due to a serious AE (SAE) not related to trial treatment. In the full analysis set, 32.1% (9/28) demonstrated sufficient IU and received RAI—the IT cohort.

Four of 9 (44.4%) IT participants and 1 of 19 (5.3%) non-IT participants received all doses of selumetinib (4200 mg). Dose reductions or omissions were largely due to AEs or toxicities (79.3%).

**FIG. 1.**
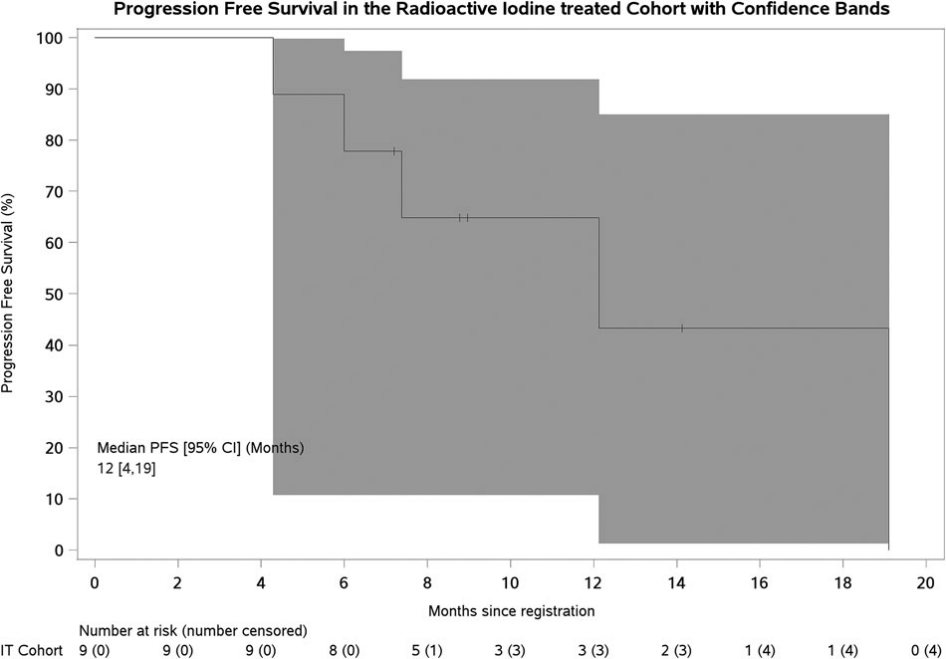
Progression-free survival with 95% (Hall–Wellner) confidence bands. CI, confidence interval; IT, iodine treated.

PFS is presented in Figure 1 (IT cohort). At analysis, 7 (77.8%) patients had progressed (2 censored at start of new treatment before progression). PFS at 12 months was 64.8% [CI: 25.3–87.2; 80% CI: 39.8–81.5].

Seven SAEs were reported in 7 participants (1 IT, 6 non-IT): 3 related to selumetinib (all non-IT; 1 pharyngeal mucositis [Grade 3], 1 international normalized ratio increased [Grade 3], and 1 creatine phosphokinase increased [Grade 4]). There were no treatment-related deaths.

All participants reported an adverse reaction relating to selumetinib. Fifty percent (14/28) reported worst CTCAE grade 1 or 2, 46.4% (13/28) grade 3, and 3.6% (1/28) grade 4.

While some patients did achieve sufficient increases in IU, the proportion was lower than anticipated. Combined with slow accrual, this led to early trial closure before reaching target sample size.

There are several possible reasons for this difference. In the previous study 12 of 20 patients had increased IU postselumetinib, the basis for our 60% prediction. However, in the pilot study only eight patients (40%) received ^131^I therapy due to the requirement that ^131^I therapy should be predicted to deliver at least 20 Gy to target lesions. Although our protocol did not set such strict targets and intended to treat any patient with a clinically relevant increase in uptake, it was perhaps optimistic to expect a conversion rate of 60%.

In SEL-I-METRY, 40% of patients had papillary carcinoma and 60% follicular carcinoma, compared with 65% papillary and 35% poorly differentiated carcinoma in the pilot study. Differing gene mutations underlying these pathologies may account for differences in outcomes. Indeed, the pilot study found this strategy less effective for tumors with BRAF mutations; only 1 of 9 of these patients received further RAI.

Different imaging modalities were also used. The pilot study used ^124^I PET/CT imaging. Due to lack of ^124^I in the United Kingdom at the time of SEL-I-METRY, a pragmatic decision was taken to use ^123^I SPECT/CT. However, we do not believe this underestimated clinically meaningful increased IU.

A further difference is the reported tolerability of selumetinib. Ho et al.^5^ reported all evaluable patients completed a full course of selumetinib without any dose reductions or delays. However, 23 of 28 of our patients required dose reduction or delay due to toxicity, with 46% experiencing grade 3 toxicity that may have contributed to the lower observed IU. Reasons for this discrepancy are unclear. There may have been differences in overall health status of treated populations, but it is not possible to determine this from the reported data.

Despite being underpowered to report the primary endpoint due to early closure, exploratory analyses showed promising clinical efficacy, with 12-month PFS 64.8% [CI: 25.3–87.2; 80% CI: 39.8–81.5] for patients who received RAI therapy. Both CIs exclude the 25% 12-month PFS assumed from historical control data. This is impressive given all patients had evidence of RECIST progression within the prior 12 months, although the limitations of nonrandomized data and potential selection of better prognosis patients must be acknowledged.

While the full clinical benefits of redifferentiation therapy are yet to be established, this is a promising approach and further studies are required. Increasing availability of more selective inhibitors of specific molecular targets, such as BRAF mutations, opens the door to a more targeted approach. There are reports of increased IU after treatment with these agents.^10^ Dosimetry data from the trial suggest that there may be opportunities to personalize RAI by adjusting administered activity based on pretherapy imaging.^11^ Attention needs to be given to different categories of RAI refractory DTC, for example, distinguishing those with absolutely no RAI uptake from those with uptake in some lesions but not all.

Given the relative rarity of RAI refractory DTC, and especially if subtypes are to be investigated, international efforts will be required to define optimal patient selection and treatment strategy, ideally through randomized phase III trials, comparing this strategy with alternative treatment approaches.
